# Identification of Novel Mt-Guab2 Inhibitor Series Active against *M. tuberculosis*

**DOI:** 10.1371/journal.pone.0033886

**Published:** 2012-03-29

**Authors:** Veeraraghavan Usha, Judith V. Hobrath, Sudagar S. Gurcha, Robert C. Reynolds, Gurdyal S. Besra

**Affiliations:** 1 School of Biosciences, University of Birmingham, Edgbaston, Birmingham, United Kingdom; 2 Drug Discovery Division, Southern Research Institute, Birmingham, Alabama, United States of America; University of Delhi, India

## Abstract

Tuberculosis (TB) remains a leading cause of mortality worldwide. With the emergence of multidrug resistant TB, extensively drug resistant TB and HIV-associated TB it is imperative that new drug targets be identified. The potential of *Mycobacterium tuberculosis* inosine monophosphate dehydrogenase (IMPDH) as a novel drug target was explored in the present study. IMPDH exclusively catalyzes the conversion of inosine monophosphate (IMP) to xanthosine monophosphate (XMP) in the presence of the cofactor nicotinamide adenine dinucleotide (NAD^+^). Although the enzyme is a dehydrogenase, the enzyme does not catalyze the reverse reaction i.e. the conversion of XMP to IMP. Unlike other bacteria, *M. tuberculosis* harbors three IMPDH-like genes, designated as Mt-*guaB1*, Mt-*guaB2* and Mt-*guaB3* respectively. Of the three putative IMPDH's, we previously confirmed that Mt-GuaB2 was the only functional ortholog by characterizing the enzyme kinetically. Using an *in silico* approach based on designed scaffolds, a series of novel classes of inhibitors was identified. The inhibitors possess good activity against *M. tuberculosis* with MIC values in the range of 0.4 to 11.4 µg mL^−1^. Among the identified ligands, two inhibitors have nanomolar *K_i_*s against the Mt-GuaB2 enzyme.

## Introduction

Tuberculosis (TB) is still a worldwide problem as the number of new cases continues to grow, approaching 9.8 million in 2010 and resulting in approximately 1.68 million deaths in 2009 [1], [2]. Human immunodeficiency virus (HIV) co-infection is a crucial factor in the rise in the number of TB cases and the development of active tuberculosis [1], [3]. In addition, multidrug resistant and extensively drug resistant strains continue to evolve, making current treatments ineffective [4], [5]. To counter the drug resistance problem there is a crucial need to identify new drug targets. Inosine monophosphate (IMP) is obtained in mycobacteria by the *de novo* purine nucleotide biosynthesis pathway wherein the purine ring is assembled in a stepwise manner starting from phosphoribosyl pyrophosphate through eleven distinct enzymatic steps [6]. IMP is a common precursor for both adenine and guanine nucleotide synthesis [7]. The first of the two steps towards guanine nucleotide biosynthesis is catalysed by inosine monophosphate dehydrogenase (IMPDH) which converts IMP to xanthosine monophosphate (XMP) with the concomitant conversion of NAD^+^ to NADH. The IMPDH reaction equilibrium strongly favors the forward reaction and maintains the guanine nucleotide pool [8]. In *M. tuberculosis* Mt-GuaB2 is solely responsible for this essential function, since out of the three genes that encode IMPDH [9] Mt-GuaB2 is the only functional ortholog [10].

IMPDH is considered an attractive target for immunosuppressive, cancer, antiviral, and antimicrobial therapy [11]. A genome wide transposon mutagenesis study indicated that *M. tuberculosis* requires Mt-GuaB2 for its survival [12], [13]. IMPDH inhibitors cause a reduction of guanine nucleotide levels and increase adenine nucleotides *in vivo*, and subsequently, DNA and RNA synthesis is interrupted resulting in cytotoxicity [11], [14], [15]. Depending on the mode of enzyme binding, IMPDH inhibitors are classified into three types: type I inhibitors are IMP/XMP analogues, type II are NAD^+^/NADH analogues and type III are multisubstrate inhibitors [16]. The first known IMPDH inhibitor was the mold metabolite mycophenolic acid (MPA) which is a type II inhibitor. MPA requires no metabolic activation and binds at the NAD^+^ site [16]. Other type II inhibitors like tiazofurin and selenazofurin must first be metabolically activated to adenine dinucleotides, thiazole-4-carboxamide adenine dinucleotide (TAD) and selenazole-4-carboxamide adenine dinucleotide (SAD) *in vivo* to become inhibitors [16]. The nucleoside analogue tiazofurin and its derivatives are uncompetitive inhibitors [6], [17], [18]. Typical type I inhibitors such as ribavirin and mizoribine bind at the substrate site [19].

MPA inhibits by trapping enzyme-XMP* (E-XMP*) as a covalent intermediate, and the pattern of inhibition is uncompetitive with respect to both the substrates IMP and NAD^+^ due to the strong preference for E-XMP* [11], [14]. MPA and mizoribine are used in immunosuppressive chemotherapy and ribavirin for antiviral chemotherapy [6], [20]. Mizoribine (MZP), an IMP analogue, is a potent inhibitor of microbial enzymes [21]. The phenyloxazole urea scaffolds were discovered in a structure-based drug design effort at Vertex Pharmaceuticals. Like MPA, these compounds trap the covalent intermediate E-XMP* complex. Imidazo[4,5-e][1,4]diazapine nucleotide is a potent inhibitor of *Escherichia coli* IMPDH [22]. Although halicyclamine was originally identified as a human IMPDH type II inhibitor, it was recently found that the antitubercular activity of halicyclamine was not due to inhibition of IMPDH [14], [23]. The first potent inhibitors of Mt-GuaB2 reported were the triazole linked mycophenolic adenine dinucleotides which showed uncompetitive inhibition with both NAD^+^ and IMP [24]. Recently, several analogues in the diphenyl urea (DPU) class of Mt-GuaB2 inhibitors were selected based on their potent antitubercular activity and informatics analysis [10].

Among the characterized bacterial IMPDH enzymes are those from *E. coli*, *Streptococcus pyogenes*, *Streptococcus suis*, *Bacillus subtilis*, *Borrelia burgdorferi*, *Halobacterium salinarum* and *M. tuberculosis* [10], [14], [24]–[29]. IMPDH exists as a homotetramer [6], [26]. Each monomer consists of two domains: the larger catalytic core domain which forms an (α,β)_8_ barrel and a smaller subdomain containing two cystathionine β synthase (CBS) domains also called the bateman domain [6], [14]. The subdomain is not required for activity although still present in all the IMPDHs characterized to date [30]. In *E. coli* the subdomain is known to regulate the distribution of adenine and guanine nucleotide pools [31]. The larger domain contains an active site loop at the C-terminal end of the β barrel strands [6], [32]. The substrates, IMP and NAD^+^ bind to the active site and, following NADH release, E-XMP* is hydrolysed [33]. During the enzymatic oxidation of IMP to XMP, the active site cysteine residue is covalently modified [6], [34].

In order to preselect for Mt-GuaB2 inhibitors that show antibacterial activity, we selected scaffolds based on whole cell antibacterial data from our previous *M. tuberculosis* H37Rv screens of three libraries: the NIH Molecular Libraries Small Molecule Repository (MLSMR) (NIH Roadmap Initiative) [35], the Life Chemicals kinase library [36] and an in house Chembridge library [37]. All compound selections were made from active compounds and full dose-response data from these screens: 2273 actives identified from the MLSMR, 1781 from the Chembridge set and 1329 from the kinase library. Only a small number of non-nucleoside, small molecule IMPDH ligands has been published for various species. We utilized core scaffolds of these known IMPDH ligands for searching our TB active sets for potential *M. tuberculosis* IMPDH inhibitors [10], [14], [38]–[46]. The search resulted in the identification of five analogues of the known IMPDH inhibitor scaffold 2-phenoxy-N-phenylpropanamide [38] and these compounds were included in the set of compounds evaluated in this study.

A focused scaffold-based approach was applied to select further compounds that also possess structural novelty as potential Mt-GuaB2 inhibitors. The *Cryptosporidium parvum* IMPDH crystal structure contains inosinate and the small molecule c46 co-crystallized in the active site [40]. We identified a structurally diverse set of small scaffolds that may be overlaid onto distinct regions of the c64 ligand as present in the crystal structure, and our *M. tuberculosis* H37Rv active sets were searched for hits that contain these substructures. Twelve such small scaffolds led to the identification of forty-three compounds among the reported TB actives, and these are shown in Figure S1. Out of the *M. tuberculosis* H37Rv actives identified based on these searches thirty-three compounds were selected for evaluation against Mt-GuaB2. These compounds include fifteen hits from the Chembridge library (Figure S2) and eighteen hits listed in Figure S3 that are available from Life Chemicals and a single compound from ChemDiv. The identified inhibitors were tested for *in vitro* antibacterial activity against *M. tuberculosis* and *M. smegmatis*, cellular cytotoxicity, and in functional Mt-GuaB2 *in vitro* assays to determine the inhibition constants and mechanism of enzyme inhibition. The target of two of the most potent Mt-GuaB2 inhibitors were identified.

## Methods

### Ethics Statement

All experimental mouse protocols and details of animal welfare and steps to ameliorate suffering were approved with written consent by the Animal Care Use Committee of Colarado State University (approval numbers ACUC # 04-302A-06 and ACUC # 06-221A-03), which abides by the USDA Animal Welfare Act and the Public Health Service Policy on Humane Care and Use of Laboratory Animals.

### Chemicals

IMP and NAD^+^ were purchased from Sigma Aldrich (St. Louis, MO, USA).

### Compound libraries

The set of thirty three small molecule compounds selected for evaluation was purchased from the commercial vendors Chembridge (Chembridge set), Life Chemicals and ChemDiv (Life Chemicals set) and were used as supplied from the vendors without further characterization or purification. The stock solutions of the test compounds were prepared in DMSO and stored at −20°C. Structures of the Chembridge set of compounds are listed in Figure S2 and the Life Chemicals set in Figure S3.

### Cuvettes

Special reduced volume UV transparent ultra micro plastibrand disposable cuvettes were obtained from Brand GmBH (Germany).

### Cloning, expression and purification of Mt-GuaB2

Mt-GuaB2 was cloned into pET28b, overexpressed in *E. coli* as a N-terminal poly histidine fusion and purified using the protocol previously described [10]. In this study 4×1L large scale cultures were grown in terrific broth supplemented with potassium salts instead of the Luria- Bertani broth.

### Mt-GuaB2 activity assay

The Mt-GuaB2 assay was carried out according to the protocol described previously except that the assay was re-standardized using a final volume of 200 µl [10]. Mt-GuaB2 activity was monitored by using a UV spectrophotometer (Jenway) and measuring the rate of formation of NADH at 340 nm for 5 minutes at 37°C. The reaction mixture (200 µl) contained 50 mM Tris.Cl pH 8.5, 150 mM KCl, 1 mM EDTA, 1 mM DTT, 1 mM IMP, 2.8 mM NAD^+^ and 3 µg of purified enzyme. The reaction was initiated by addition of substrate, IMP or NAD^+^ after preincubation of the other reaction components for 5 min at 37°C. The concentrations of IMP or NAD^+^ were varied for *K_m_*_ (app)_ determination at saturating concentrations of the other substrate and the initial rate data were fitted to the Michaelis Menten equation using Graphpad prism software. For determination of the *K_m_*_ (app)_ value for IMP, the concentration of IMP in the reaction mixture was varied from 0.010–1.5 mM at saturating concentrations of NAD^+^ (2.8 mM). A minus IMP control was included. Initial rate data were also generated using a fixed IMP concentration (1.086 mM) while varying NAD^+^ concentrations (0.031–3.0 mM). The slope was determined from the linear portion of the reaction time course and plotted against IMP or NAD^+^ concentration (µM).

### Inhibitor kinetics

The *K*_i_ value for NAD^+^ was determined at a constant saturating IMP concentration (1.086 mM, *K_m_* IMP 108.6 µM) and three different concentrations of NAD^+^ (0.35, 0.70 and 1.4 mM) in the presence of increasing concentrations of inhibitor (0.01–1000 µM). The value of *K*_i_ for IMP was determined at fixed saturating concentration of NAD^+^ (2.8 mM, *K_m_* NAD^+^ 699.4 µM) and different concentrations of IMP (0.054, 0.108 and 0.217 mM) and inhibitor (0.01–1000 µM). Mt-GuaB2 was pre-incubated with varying inhibitor concentrations for five minutes at 37°C prior to addition of IMP or NAD^+^ to start the reaction, and the samples were assayed for remaining enzyme activity over a five minute period. The inhibitors were dissolved in 100% DMSO and diluted to a final concentration of 1% in experimental reactions, i.e. 2 µl of the inhibitor stock was used in a 200 µl enzyme assay. In control samples, compounds were replaced with the same volume of neat DMSO. The initial velocities at various inhibitor concentrations were determined based on the slope in the linear part of each reaction containing the inhibitor and the uninhibited reaction. To determine the inhibition constant (*K*_i_ values) the initial rate data *versus* substrate concentration at different inhibitor concentrations were fit using the program Prism version 5.0 (GraphPad San Diego, CA) to equations for uncompetitive, competitive, or noncompetitive inhibition. For each inhibitor concentration, the reciprocal of enzyme reaction velocity *versus* the reciprocal of the substrate concentration was plotted in a Lineweaver-Burk plot to determine the pattern of inhibition.

### Drug susceptibility testing of wild type *M. smegmatis*, recombinant Mt-GuaB2 cloned in pVV16 and wild type *M. tuberculosis*

The susceptibility of *M. smegmatis* cells overexpressing the Mt-GuaB2 plasmid or empty pVV16 vector control was determined in the presence of increasing drug concentrations (compounds 5217501, 6655281 and 7759844) as described previously for the diphenyl ureas (DPU's) [10]. Each compound was freshly dissolved in DMSO at a concentration of 10 mg/ml. The final concentration of DMSO in the MIC assays was 1%. To determine the minimum inhibitory concentration (MIC) of wild type *M. smegmatis* the cultures were grown to an OD_600_ of 0.7, serially diluted ten fold (10^−1^ to 10^−4^) and spotted (5 µl) on tryptic soy broth (TSB) agar plates containing two fold serial dilutions of the compound (0.25–128 µg/ml) or an untreated control (neat DMSO). The plates were incubated at 37°C for 3 days. The MIC is defined as the minimal concentration required to completely inhibit 99% of mycobacterial growth. All evaluated compounds were previously screened for activity against *M. tuberculosis* using a whole cell microplate Alamar blue assay [35]–[37].

### Determination of bactericidal activity of compounds against *M. smegmatis*

*M. smegmatis* was grown in MB7H9 broth containing 0.2% (v/v) glycerol, 0.05% Tween 80 and 10% ADC enrichment till early logarithmic phase (OD_600_ of 0.8) and the cells were subsequently diluted to an OD_600_ of 0.035 (∼1×10^6^ cfu/ml) with MB7H9 broth. Diluted bacterial cultures (3 ml aliquots) were added to each tube containing the two fold dilutions of compound 5217501 (1.5–48 µg/ml), 6655281 (0.75–100 µg/ml) and 7759844 (0.5–32 µg/ml) and the control tubes containing neat DMSO and were incubated with continuous agitation at 180 rpm and 37°C for 24 hours. Post incubation, the cultures (0.1 ml) were serially diluted ten fold (10^−1^ to 10^−6^) with MB7H9 broth and the bactericidal effect of compounds determined by plating 0.1 ml volumes of 10^−4^, 10^−5^ and 10^−6^ dilutions on MB7H10 agar plates containing 0.5% glycerol and 10% OADC enrichment after incubation at 37°C for 3 days. The resulting bacterial colonies were counted. Percent inhibition is defined as number of colonies in the compound treated tubes divided by the number of colonies in untreated tubes, times 100%. The experiment was done in duplicate and the mean and standard error determined. The minimum bactericidal concentration (MBC) is defined as the minimal concentration which effectively reduced at least 99% of the viable counts in the compound treated sample compared with untreated samples.

### Modeling Mt-GuaB2–IMP complex structure

Mt-GuaB2 was modeled based on the human IMPDH type II crystal structure (PDB code 1NFB), utilizing a previously published sequence alignment [10]. The sequence identity between Mt-GuaB2 and the human type II IMPDH template in the modeled region is 49.51%, well above the ∼30% threshold typically considered acceptable for homology modeling. The initial homology model was relaxed through restrained energy minimization using Impact tools from the Protein Preparation Refinement module of the Schrodinger software package. The substrate IMP was modeled into the active site of Mt-GuaB2, and taken from the *Cryptosporidium parvum* IMPDH crystal structure (PDB code 3KHJ) after superimposing the latter structure onto the Mt-GuaB2 model. After the initial energy minimization of the obtained protein–IMP complex, the protein part was replaced by the structure prior to energy minimization, and the system re-minimized with restrained energy optimization in Impact (Schrodinger software). This approach allowed an initial adjustment of IMP prior to relaxing the protein structure together with IMP. The structure of IMP in the obtained Mt-GuaB2–IMP model was close to that of the co-crystallized purine riboside phosphate present in the human type II IMPDH (1NFB) structure; slight deviations between these two related ligands are limited to the purine ring, which is covalently linked to the protein in the human type II IMPDH crystal structure but not in the Mt-GuaB2-IMP model. Residues that participate in direct interactions with either ligands in these two complex structures are conserved between the two except for the conservative replacement of R322 in the human type II IMPDH by K332 in Mt-GuaB2.

### *In vivo* studies

#### Maximum tolerated dose (MTD) test

Concurrent with the advanced enzymatic screens, compound 7759844 was selected for further evaluation *in vivo* due to its excellent activity against *M. tuberculosis* H37Rv and strong inhibition of Mt-GuaB2. Both toxicity and efficacy in a murine *M. tuberculosis* model were undertaken in order to evaluate these scaffolds as further candidates for antitubercular drug discovery against IMPDH. In two separate studies, the tolerability of 7759844 was studied in an acute maximum tolerated dose C57BL/6 mouse model (MTD) to evaluate toxicity and determine a safe dose in the murine TB model. Mice were administered a single dose of compound formulated in 0.5% methyl cellulose at three doses (100, 300, and 500 mg/kg) by gavage followed by observation for seven days. There were no dose related adverse effects seen through the highest dose (500 mg/kg). Subsequently, 7759844 was tested for efficacy in GKO mice.

### Animal efficacy screening

Once an acceptable MTD value was determined, the compounds were tested for *in vivo* efficacy in a short term mouse *M. tuberculosis* model. This approach uses the interferon-γ gene-disrupted C57BL/6 mice (GKO) in a murine TB aerosol infection model developed and extensively tested by Lenaerts *et al* [47]. GKO mice do not mount an adaptive immune response and any antitubercular efficacy seen can be exclusively attributed to drug action [48], [49]. GKO mice were infected with *M. tuberculosis* Erdman strain by a low dose aerosol (LDA) exposure using a MB aerosol generation device [47]. GKO mice are highly susceptible to *M. tuberculosis* infection and the short term mouse model required only eight days of treatment after fifteen days of infection [50], allowing the rapid *in vivo* assessment of candidate drugs for antitubercular activity. Twelve week old female GKO mice obtained from Jackson laboratories were used in this study. After fifteen days of infection, mice were treated by oral gavage with 7759844 for eight consecutive days until the twenty-third day and sacrificed on the twenty-fourth day. Each group consisted of five mice. The first group received 300 mg/kg body weight of 7759844, the second group received the positive control isoniazid at 25 mg/kg body weight and the third group was the untreated negative control. Compound 7759844 was suspended in 0.5% methyl cellulose and isoniazid was dissolved in sterile water. At the end of the experiment the lung and spleen homogenates of untreated and drug treated mice were cultured and the viable colonies were counted after four weeks of incubation at 37°C. The viable counts were converted to logarithms and the mean of 7759844 was compared with the untreated control by one way analysis of variance (ANOVA) followed by Dunnett's post-test.

## Results

### Purification of Mt-GuaB2

Recombinant Mt-GuaB2 was overexpressed in *E. coli* and the enzyme purified by a single step HisTrap Ni^2+^ chelating Sepharose affinity chromatography. SDS-PAGE showed a distinct band of Mt-GuaB2 at an apparent molecular weight of approximately 57 kDa (Figure S4). The fractions of highest purity were retained, pooled, dialysed, concentrated and used for the activity assays.

### Determination of *K_m_*_ (app)_ for substrates

The assay volume was reduced to 200 µl from 1 ml and the kinetic constants reassessed as reported [10]. The Mt-GuaB2 activity was examined with variable concentrations of substrate and a fixed saturating concentration of co-substrate to determine the kinetic constants. The *K_m_*_ (app)_ of IMP and NAD^+^ were determined to be 108.6+/−11.97 µM and 699.4+/−63.70 µM respectively (Figure S5). The *K_m_*_ (app)_ of IMP was lower and that of NAD^+^ higher than that reported earlier [10]. Compared to mammalian IMPDHs, bacterial IMPDH enzymes tend to bind IMP more effectively and bind NAD^+^ with a low affinity [26]. The pathogenic bacterium *S. pyogenes* IMPDH has kinetic characteristics similar to other bacterial IMPDH enzymes, its *K_m_* value for NAD^+^ is on the ‘high’ end, approximately 1180 µM and its *K_m_* for IMP on the ‘low’ end, 62 µM [29], [34].

### Screening of the Chembridge and Life Chemicals sets against purified Mt-GuaB2 and determination of *K*_i_ values

**Figure 1:**
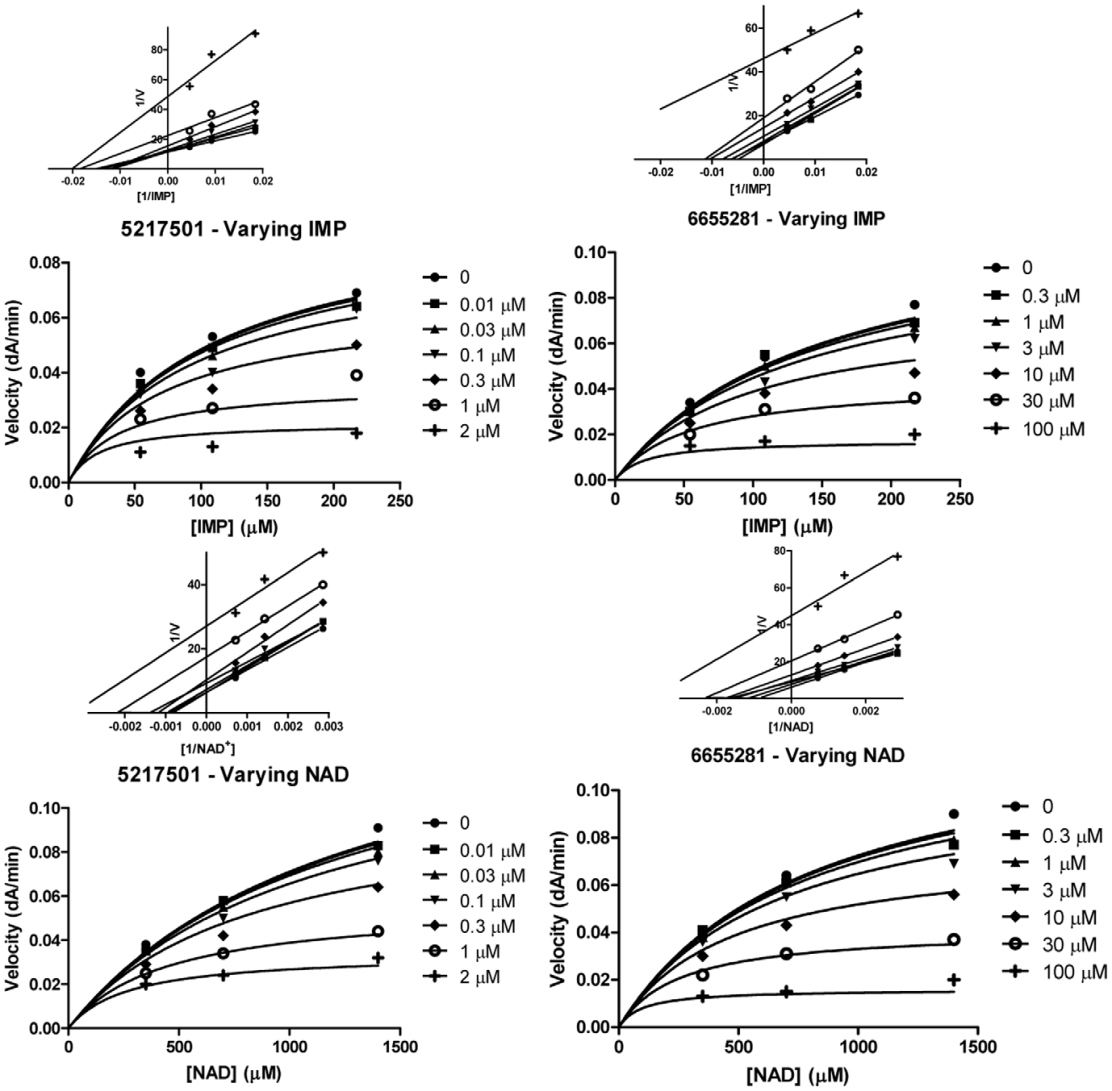
Mechanism of inhibition graphs of Chembridge compounds 5217501 and 6655281 against substrates IMP and NAD^+^. The recombinant Mt-GuaB2 enzyme from *E. coli* was assayed as described in Materials and Methods in the absence of inhibitor and increasing concentrations of compounds 5217501 and 6655281. IMP and inhibitor were used at varying concentrations (Top panel), the reaction was started by addition of a fixed saturating concentration of NAD^+^ and the OD measured at 340 nm. In the bottom panel a variable concentration of NAD^+^ and inhibitor was used and a fixed saturating IMP concentration to initiate the reaction. The velocity data were fitted in Graphpad prism by a nonlinear regression method. Compounds were assayed in duplicate and the data represent the mean +/− standard error of two independent experiments. The 1/V versus 1/[Sub] plot at the inset (top) of each graph was generated to indicate the pattern of inhibition.

**Figure 2:**
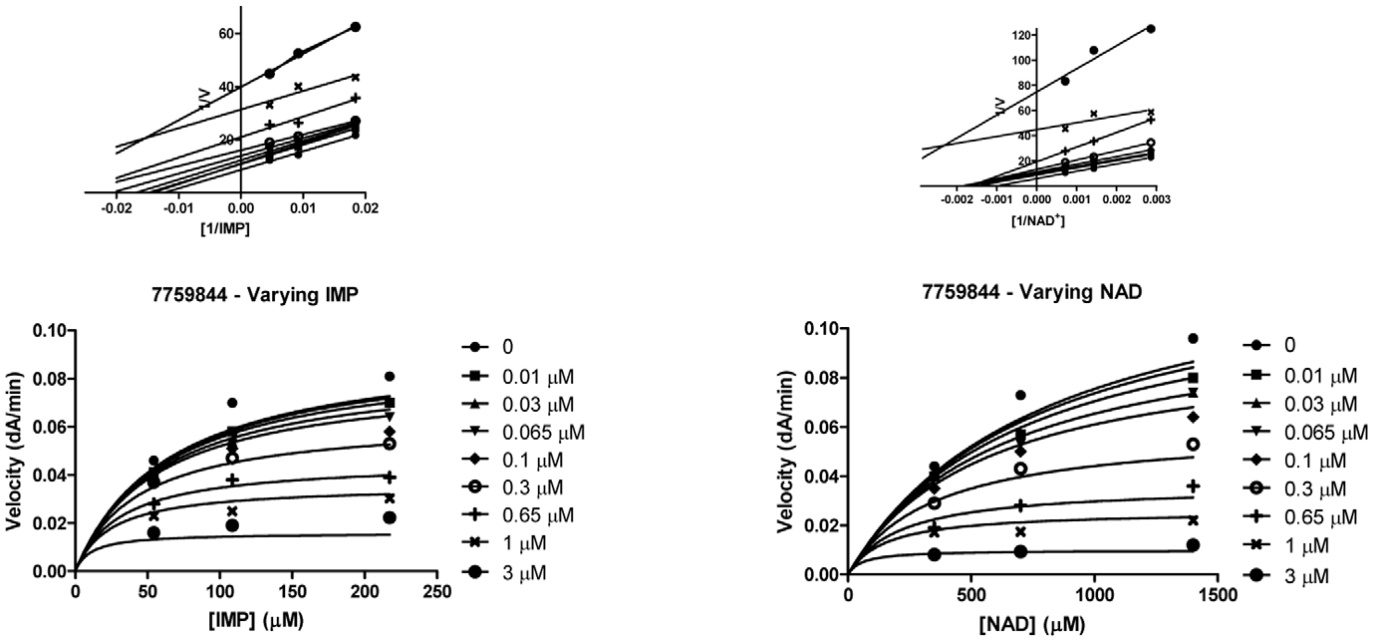
Mechanism of inhibition of Chembridge compound 7759844 against both substrates IMP and NAD^+^. The purified Mt-GuaB2 enzyme was incubated at 37°C for five minutes with substrate in the absence and presence of different concentrations of compound 7759844 and the reaction started by addition of NAD^+^ or IMP. The initial velocity data were plotted against three different IMP (left panel) or NAD^+^ concentrations (right panel) and varying inhibitor concentrations. The data represent the mean +/− standard error of duplicate experiments. The 1/V versus 1/[Sub] plot at the inset (top) of each graph only depicted the type of inhibition.

A selected set of commercially available compounds from the Chembridge and the Life Chemicals libraries were evaluated for their inhibition in the spectrophotometric assay using purified Mt-GuaB2. Inhibition constants, *K*_i_ values, with respect to both substrates IMP and NAD^+^ were determined by assaying various concentrations of the inhibitor in the presence of three different concentrations of substrate and a fixed saturating concentration of the co-substrate. The inhibition data for the fifteen Chembridge compounds are summarized in Table 1. Almost eleven compounds in this set were found to inhibit Mt-GuaB2 with *K*_i_ values lower than 100 µM and four hits bolded in Table 1 have low micromolar inhibition constants. The most potent compounds 5217501 and 7759844 have *K*_i_ values in the nanomolar range with respect to both substrates IMP and NAD^+^ and also inhibit *M. tuberculosis* and *M. smegmatis* growth. The analogue 6655281 shows low micromolar inhibition, substantial anti-mycobacterial activity against *M. tuberculosis* and it was the most potent compound in this series against *M. smegmatis* growth. All compounds excepting 7577905 yielded an uncompetitive inhibition pattern with respect to IMP and NAD^+^. Figures 1 and 2 illustrated the pattern of inhibition of the three Chembridge hits with low micromolar *K*_i_. *K*_i_ values were determined from nonlinear regression analysis in GraphPad prism by fitting data to the equation for uncompetitive inhibition.

**Table 1:** α*K*_i_ (µM) values of Chembridge compounds against IMP and NAD^+^, *in vitro* activity of Chembridge compounds against *M. tuberculosis* H37Rv and *M. smegmatis* and selectivity index (SI).

ChemBridge I.d.	IMP *K*_i_ (µM)	IMP α*K*_i_ (µM)	NAD *K*_i_ (µM)	NAD α*K*_i_ (µM)	*M. tb* MIC_90_ (µg/ml)	SI	*M. smeg* MIC_90_ (µg/ml)
5217501		**0.548 (UC)**		**0.523 (UC)**	4.83	>8.76	**12**
6655281		**16.7 (UC)**		**13.9 (UC)**	3.45	3.66	**0.75**
7240597		101.6 (UC)		94.1 (UC)	10.43	NA	40
7383310		36.9 (UC)		95.8 (UC)	5.50	>2.73	20
7407367		142.3 (UC)		141.3 (UC)	7.45	>2.01	>28
7409994		**6.46 (UC)**		**3.36 (UC)**	3.27	>4.59	>128
7577905	243.6 (NC)		151.4 (C)		2.35	>6.37	48
7610600		34.6 (UC)		29.2 (UC)	1.65	>9.11	>96
7622922		136.5 (UC)		804.8 (UC)	1.84	>8.14	>128
7741101		42.9 (UC)		72.1 (UC)	5.47	>7.31	>128
7741703		24.7 (UC)		24.8 (UC)	3.29	>4.56	48
7756985		96.6 (UC)		207.4 (UC)	0.80	>18.66	4
7759844		**0.603 (UC)**		**0.233 (UC)**	0.63	>23.70	**8**
7913222		126.0 (UC)		92.3 (UC)	6.27	>2.39	64
9008726		31.0 (UC)		44.0 (UC)	4.26	>15	NA

SI defined as 
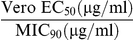
 i.e. the ratio of vero cell cytotoxicity to the anti-TB activity.

Note: (UC) stands for Uncompetitive inhibition, (NC) Noncompetitive, (C) Competitive, (NA) Not available and (SI) Selectivity index.

Table 2 listed the activity and inhibition constant data for the Life Chemicals compound set, consisting of seventeen compounds from Life Chemicals and one from ChemDiv. Compound F1374-1083 was the best inhibitor in this set with low micromolar *K*_i_, and also inhibited *M. tuberculosis* growth but did not inhibit *M. smegmatis* growth even at the highest concentration tested (128 µg/ml). All except for F1374-0253 were uncompetitive inhibitors of the enzyme with respect to the substrate NAD^+^, and all these compounds showed uncompetitive inhibition with respect to IMP.

**Table 2:** Summary of Mt-GuaB2 inhibition constants of Life Chemicals compounds, MIC values of Life Chemical compounds against *M. tuberculosis* and *M. smegmatis* and selectivity index (SI) values.

Life Chemicals I.d.	IMP α*K*_i_ (µM)	NAD *K*_i_ (µM)	NAD α*K*_i_ (µM)	*M. tb* MIC_90_ (µg/ml)	SI	*M. smeg* MIC_90_ (µg/ml)
F1374-0033	60.9 (UC)		45.0 (UC)	1.75	7.89	8
F1374-0205	35.3 (UC)		100.0 (UC)	5.59	3.43	>128
F1374-0253	11.2 (UC)	434.6 (NC)		5.30	>7.55	>128
F1374-0873	35.5 (UC)		47.3 (UC)	5.84	>6.85	>128
F1374-0885	19.0 (UC)		28.2 (UC)	0.72	6.55	2
F1374-0978	28.3 (UC)		31.8 (UC)	2.98^a^	4.6^a^	>128
F1374-1083	**4.86 (UC)**		**6.13 (UC)**	4.81	>8.31	>128
F1499-0691	404.2 (UC)		273.9 (UC)	NA	NA	4
F1685-0077	229.3 (UC)		275.6 (UC)	6.88	>5.81	>128
F2517-0198	19.1 (UC)		64.9 (UC)	2.68	3.84	>128
F2518-0183	57.1 (UC)		73.5 (UC)	1.76^a^	17^a^	>128
F2518-0274	765.4 (UC)		895.3 (UC)	5.50	5.11	20
F2518-0386	22.6 (UC)		55.1 (UC)	0.41^a^	>96.85^a^	>128
F2518-0401	501.9 (UC)		591.0 (UC)	1.18	12.51	16
F2518-0413	36.4 (UC)		52.7 (UC)	1.23	6.67	>128
F2518-0418	328.7 (UC)		478.2 (UC)	2.24	8.20	32
F2518-0484	35.5 (UC)		71.3 (UC)	6.70	>5.97	3
F2536-1573	79.0 (UC)		55.0 (UC)	1.02	6.68	>128

Compound 1499-0691 from ChemDiv is also included in this set.

Note: (UC) stands for Uncompetitive inhibition, (NC) Noncompetitive, (NA) Not available and (SI) Selectivity index.

a *M.tb* MIC_90_ (µg/ml) and SI values cited from Reynolds *et al.*, 2011.

### Evaluation of compounds for antimycobacterial activity

The Chembridge and Life Chemicals sets were assessed for *in vitro* anti-mycobacterial activity against *M. tuberculosis* and *M. smegmatis*. Only compounds that have shown significant antimycobacterial activity against *M. tuberculosis* H37Rv in our previous screens [35]–[37] were included among compounds selected for the evaluations shown in Tables 1 and 2. For Chembridge compounds (Table 1), the *M. tuberculosis* MIC_90_'s range from 0.63–10.43 µg/ml. The most potent compounds included analogues of the 2-phenoxy-N-phenylpropanamide scaffold [38] such as 7759844, 7756985, 7409994, and the novel structures 7577905, 9008726, 5217501, 7383310 (for structures see Figure S2). The most potent Chembridge compound against *M. smegmatis* was 6655281 with a MIC_90_ of 0.75 µg/ml. It was structurally similar to reported IMPDH inhibitor scaffold [38]. Furthermore, two analogues (7759844 and 7756985) of this known scaffold were effective at inhibiting *M. smegmatis* growth. A structurally novel inhibitor, 5217501, was effective against both *M. tuberculosis* and *M. smegmatis*, showing MIC_90_ values of 4.83 and 12 µg/ml respectively. The selectivity index (SI) was calculated taking the ratio of the mammalian cell cytotoxicity (EC_50_) to the concentration of compound needed to inhibit bacterial growth by 90% (SI = EC_50_/IC_90_), and the reported selectivity index for 5217501 was greater than 8.76 [37]. The hits with the best reported SI values in Table 1 were 7759844 and 7756985, SI>23.70 and >18.66, respectively [37]. These two compounds were also potent inhibitors of both *M. tuberculosis* and *M. smegmatis*.

Of the eighteen hits from the Life Chemicals and ChemDiv compound set, seven compounds exhibited antimycobacterial activity against *M. smegmatis*, four of which possessed MIC_90_'s≤8 µg/ml (Table 2). *M. tuberculosis* MIC_90_'s were less than 6.88 µg/ml for all compounds (except for one untested ChemDiv compound, 1499-0691). The structurally novel compound F1374-1083 was the most potent (a low micromolar inhibitor in the enzyme assay) although it was inactive up to 128 µg/ml against *M. smegmatis*. F2518-0386 was the most potent *M. tuberculosis* growth inhibitor in this set with an MIC_90_ of 0.41 µg/ml and a high selectivity index (SI >96.85) although it was also inactive against *M. smegmatis*.

### Effects of three Chembridge compounds on viability of *M. smegmatis*

In order to determine whether selected (MIC values ≤12 µg/ml) Chembridge compounds (5217501, 6655281 and 7759844) were bactericidal or bacteriostatic against *M. smegmatis*, colonies were enumerated after 24 hours exposure to the compounds at different concentrations after plating 100 µl of diluted bacterial suspension on MB7H10 agar plates. For reference, an untreated control was included and the bactericidal effect of the compounds was compared between treated and untreated cultures.

**Figure 3:**
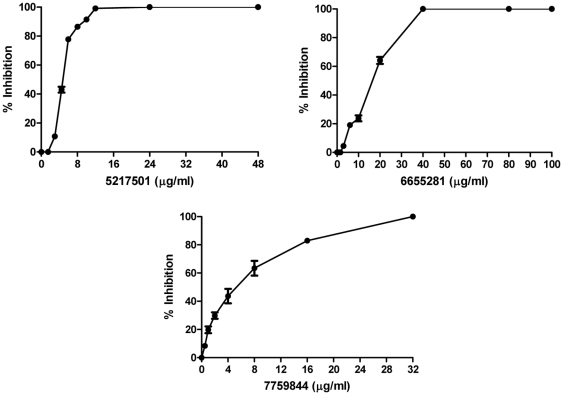
Effect of three Chembridge compounds on the viability of *M. smegmatis*. Early log phase *M. smegmatis* culture was adjusted to 1×10^6^ cfu/ml with MB7H9 broth and the culture incubated in absence (DMSO) and presence of various concentrations of inhibitor (5217501, 6655281 and 7759844) at 37°C for 24 hours. A 100 µl aliquot of 24 hour culture was serially diluted, plated on MB7H10 agar plates and incubated at 37°C for 3 days. The number of bacterial colonies in compound treated tube divided by the number of colonies in untreated tube, times 100% gave the % inhibition values which were plotted against varying inhibitor concentrations. The mean +/− standard error values of the experiment done in duplicate were plotted.

All three compounds reduced colony forming units of *M. smegmatis* culture in a dose dependent manner when compared to the untreated control indicating that the compounds were bactericidal. The kill curves for the three compounds are shown in Figure 3. The most effective compound against *M. smegmatis* was 5217501 which showed 99% inhibition of bacterial growth at 12 µg/ml and complete inhibition of growth at higher concentrations. The minimum bactericidal concentration (MBC) values obtained for compounds 6655281 and 7759844 were 40 µg/ml and 32 µg/ml, respectively.

### Investigation of the mode of action of selected Chembridge compounds

**Figure 4:**
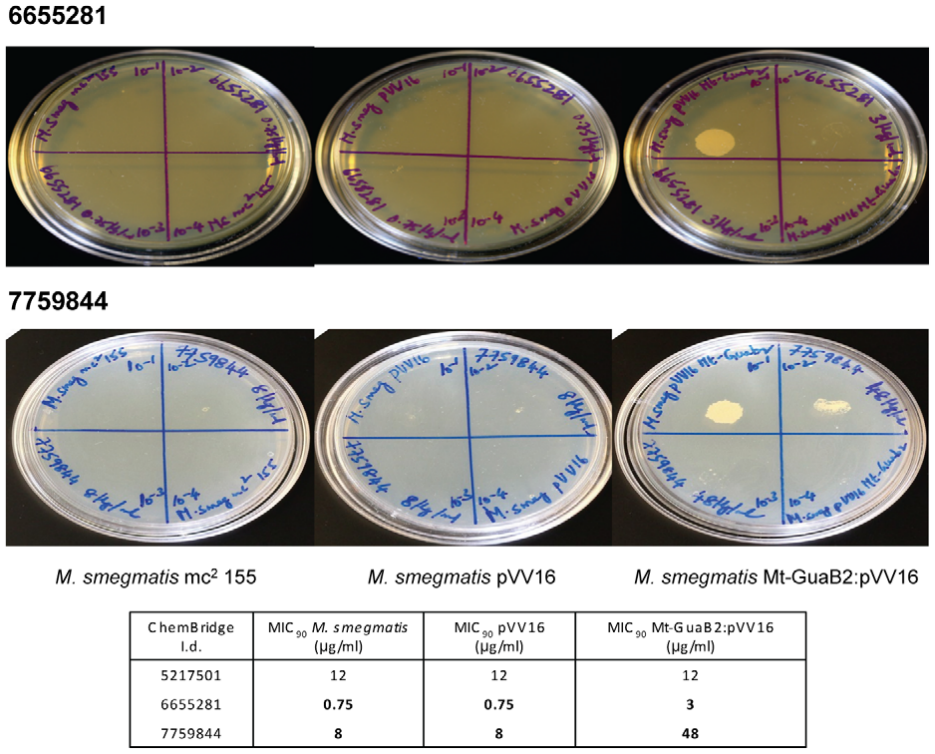
Determination of MIC (µg/ml) of three Chembridge compounds against *M. smegmatis* overexpressing Mt-GuaB2, *M. smegmatis* pVV16 and *M. smegmatis* mc^2^ 155 to ascertain the mode of action. *M. smegmatis* mc^2^ 155 (left plate), *M. smegmatis* pVV16 (middle plate) and Mt-GuaB2 overexpression construct in pVV16 (right plate) were grown to mid log phase and serial dilutions of cultures (10^−1^ to 10^−4^) were spotted on TSB plates in the absence and presence of different concentrations of compounds 6655281 and 7759844. The plates were incubated at 37°C for 3 days. The minimal concentration of the inhibitor which completely inhibited 99% of *M. smegmatis* growth was considered as MIC. The MIC results of compound 5217501 are shown in the table below.

Three selected Chembridge compounds (5217501, 6655281 and 7759844) inhibited Mt-GuaB2; their *K*_i_ values were in the low micromolar range. For the mode of action (MOA) studies, the MIC's of the Mt-GuaB2 overexpressing strain in pVV16 was determined and compared with the MIC's of the vector alone control (pVV16) and wild type *M. smegmatis*. The Mt-GuaB2 overexpressing strain showed a four fold increase in MIC for compound 6655281 and a 6 fold increase in MIC for compound 7759844 which indicated that these two compounds inhibited *M. smegmatis* growth due to the inhibition of Mt-GuaB2 (Figure 4). Hence, the target of these two inhibitors appeared to be Mt-GuaB2. The possibility of other additional unknown targets for these inhibitors cannot be ruled out and might exist. In the case of compound 5217501, the MIC's obtained for the Mt-GuaB2 overexpressing strain was similar to those of the pVV16 vector control and wild type *M. smegmatis* which suggested that compound 5217501 did not primarily target Mt-GuaB2 (Figure 4) in the bacterium.

### Docking lead inhibitor into Mt-GuaB2 and selectivity considerations against human IMPDH type II

**Figure 5:**
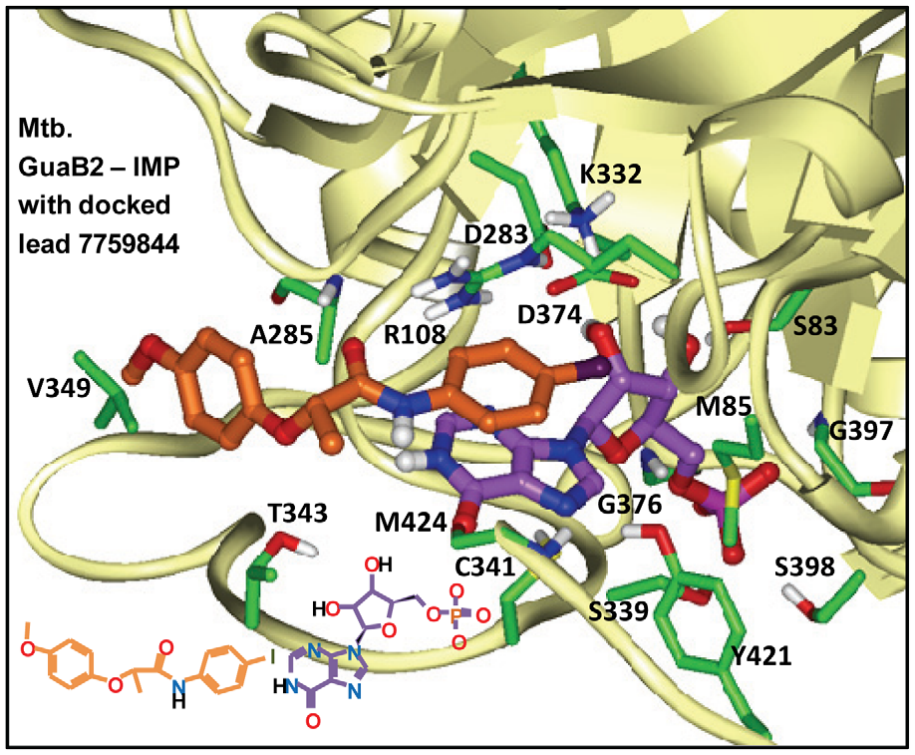
Mt-GuaB2–IMP model structure with docked lead 7759844. Close-up view of the docked lead 7759844 and IMP at the substrate binding region of the Mt-GuaB2 model. Amino acids mapping the ligand/substrate binding site are shown. Atoms are colored by type (oxygens red, nitrogens blue, sulfur yellow, polar hydrogens white). Carbon atoms of the enzyme, IMP and lead 7759844 are colored green, purple and light brown, respectively. The inset shows the 2-D structures of the ligand and IMP.

**Figure 6:**
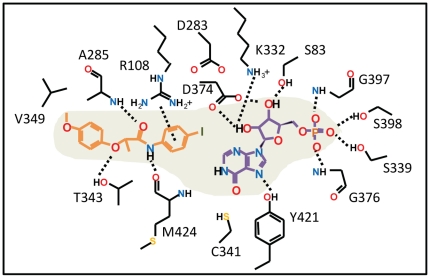
2-D representation of predicted interactions between lead 7759844 and the Mt-GuaB2–IMP structure. This 2-D representation corresponds to the Mt-GuaB2-IMP model and docked ligand shown in Figure 5, illustrating residues that interact with lead 7759844. Polar interactions are indicated with dashed lines.

Compound 7759844 was docked into the Mt-GuaB2–IMP complex structure using the InducedFit docking protocol from the Schrodinger software package that treated residues within 5 Å of docked ligand conformers as flexible. Two possible docked orientations were predicted for this ligand, one showed excellent consistency with the limited SAR data available for this lead scaffold, as listed in Table 3. Therefore, we considered this mode as our working model of 7759844 binding to Mt-GuaB2, for further validation. Our working ligand binding pose of 7759844 is shown in Figure 5 and as a 2-D illustration in Figure 6. Figures 5 and 6 illustrated the docked pose of the (*S*)-enantiomer. The terminal 4-iodophenyl group of the ligand forms an optimal aromatic stacking interaction with IMP and interacts favorably with the positively charged guanidinium moiety of R108. Interestingly, the same interaction could not form in case of the human type II IMPDH where this Arg was replaced by H93. Although the imidazole ring of a His could form favorable interactions with the phenyl ring, its shorter side chain would place the imidazole beyond an optimal distance. Any change or deviation in the position of the phenyl ring from that of the optimal for aromatic stacking with IMP would likely result in a loss of binding. This prediction is also supported by the limited SAR data available (Table 3). The dramatic reduction of activity upon introducing an *ortho*-Me on this ring (and replacing I with Cl) may be due to the effect of perturbing the phenyl position from the optimal stacking interactions with IMP. In the docked structure, the distance between the phenyl's 2-carbon and the closest backbone atom (of M424) was only 3.7 Å, and therefore, a methyl substituent could not be accommodated at this position without perturbing the phenyl ring and its stacking interaction with IMP. Rotating the ring in the docked structure would cause the *ortho*-Me to clash with IMP. The amide carbonyl of 7759844 in the docked pose was hydrogen bonded with the backbone of A285 while the Ala side chain was positioned at 3.9 Å distance from the terminal phenyl ring. In the human IMPDH sequence A285 corresponded to S276 and, considering the analogous pose bound to the human IMPDH, Ser replacing Ala would be less favorable at such close distance. The docked pose of 7759844 also suggested that favorable non-polar and hydrogen bonding interactions with T343 would favor the *S* enantiomer as opposed to the *R* enantiomer while prediction showed consistency with the inhibition data previously reported for the enantiomers of a close analog [46]. In the environment surrounding the ligand's 4-OMe-phenyl group, V345 and H286 corresponded to E335, Q277, respectively, in the human sequence. Although these residues have no direct contacts with the docked 7759844 inhibitor, they might be explored by other analogs or other compounds for improvement of selectivity against human IMPDH. Note that the evaluated sample of 7759844 was a racemic mixture, and both enantiomers were used for docking.

**Table 3:** Analogs of lead scaffold from Figure S2 docked into Mt-GuaB2 model.

ChemBridge I.d.	IMP α*K*_i_ (µM)	*M. tb* MIC_90_ (µg/ml)
7759844	0.603	0.633
7756985	96.64	0.804
7240597	101.6	10.43
7407367	142.3	7.447
7409994	6.458	3.266

### Animal studies

The efficacy of compound 7759844 was tested in an *in vivo* short term mouse infection (GKO) model. Prior to efficacy testing the compound was examined for toxicity in normal C57BL/6 mice and the compound 7759844 was well tolerated with no acute adverse effects observed at the highest single dose of 500 mg/kg. Efficacy of the compound was evaluated by administering daily doses of 7759844 at 300 mg/kg body weight for eight consecutive days *via* oral gavage to GKO mice beginning on day fifteen of infection. Even at a dose under the MTD for C57BL/6 mice, 7759844 caused distress in infected interferon-γ gene-disrupted C57BL/6 mice (GKO), and three animals were sacrificed after eight days of dosing. The compound was inactive and did not reduce the bacterial load in lungs, but it showed slight activity in the spleen, and reduced the bacterial load by 0.84 log_10_ protection units when compared to the untreated controls (Table S1 and Figure S6). Isoniazid reduced the number of colony forming units by 2.7 log_10_ protection units in lungs and 4.0 log_10_ protection units in the spleen *versus* the drug free control (Table S1 and Figure S6).

## Discussion

The potential of IMPDH for immunosuppressive, cancer and antiviral chemotherapy has been previously explored. Recently, Mt-GuaB2 has gained attention as a promising anti-mycobacterial target, associated with a number of distinct inhibitor scaffolds [10], [24]. The applicability of available bacterial IMPDH inhibitors may be limited by toxicity issues as there is an important human homolog, and lead optimization must focus on selectivity as well as potency. In the search for new lead compounds as potential Mt-GuaB2 inhibitors, we employed a designed scaffold-based approach utilizing the IMPDH crystal structure with c64 co-crystallized in the active site (PDB code 3KHJ). *M. tuberculosis* H37Rv active sets from our previous screens [35]–[37] were searched based on a set of designed scaffolds listed in Figure S1 as well as a known IMPDH scaffold [38]. Of the identified *M. tuberculosis* actives, we evaluated thirty-three compounds for their potential to inhibit Mt-GuaB2 functional activity and for their inhibitory potency against *M. smegmatis*.

In the present study, the assay was adapted to a 200 µl volume, conditions were optimized by varying concentrations of the substrate IMP, the cofactor NAD^+^ and the enzyme, and the *K_m_*_ (app)_ were determined for IMP and NAD^+^. The optimized assay yielded a reaction that proceeded linearly over a 5 min period. Next, we studied the inhibition of Mt-GuaB2 by a series of commercially available compounds, primarily from the Chembridge and Life Chemicals libraries. When compared to the DPU's, these compounds show an improvement in enzyme inhibitory activity, yielding low micromolar inhibitors. The *K*_i_ values were obtained for all inhibitors with respect to both substrates IMP and NAD^+^, and the patterns of inhibition were inferred from the graphs. Among the Chembridge compounds, four (5217501, 6655281, 7409994 and 7759844) exhibited low micromolar affinity *K*_i_ values. Out of the Life Chemicals compound set, F1374-1083 showed a low micromolar inhibition constant. All inhibitors from the Chembridge compound set (except for 7577905) showed an uncompetitive pattern of inhibition against both substrates. Uncompetitive inhibition has been observed previously *versus* both IMP and NAD^+^, in the case of compounds having a strong preference for the E-XMP* complex [14]. The observation of an uncompetitive pattern of inhibition also depended on assay conditions [51]. In the case of uncompetitive inhibition, both the substrates bind to the enzyme before the inhibitor binds. The uncompetitive inhibitors have an advantage as drugs since inhibition increased due to accumulation of substrates [52].

Except for compound F1374-0253, all compounds from the Life Chemicals set showed uncompetitive inhibition with respect to both IMP and NAD^+^. Well known uncompetitive inhibitors are MPA, an NAD^+^ mimic, SAD (which is an analog of tiazofurin metabolite TAD) and VX-497 [6], [17]. Uncompetitive inhibitors bind to the cofactor NAD^+^ binding portion of the active site [25]. MPA does not structurally resemble either the substrates or products of the IMPDH reaction. Like MPA, most uncompetitive inhibitors trap E-XMP* [51]. Life Chemicals compound F1374-0253 shows uncompetitive inhibition *versus* IMP and noncompetitive inhibition *versus* NAD^+^. Such a pattern of inhibition is exemplified by thiazole-adenine-dinucleotide (TAD) [51].

The minimum bactericidal concentration and mode of inhibition has been evaluated for three promising Chembridge compounds, 5217501, 6655281 and 7759844 that potently inhibit Mt-GuaB2 activity and show substantial inhibition of *M. smegmatis* growth *in vitro*. Compound 7759844 has the 2-phenoxy-N-phenylpropanamide core structure of a known IMP dehydrogenase inhibitor scaffold [38] and may be considered a potent analogue structure (Figure S2). Compound 6655281 also shares some structural similarities to the latter scaffold, while the inhibitor 5217501 structure is novel. IMPDH is a cellular target of compounds 6655281 and 7759844, although additional cellular targets cannot be ruled out. If the pathway is blocked by an IMPDH inhibitor DNA and RNA synthesis will be inhibited resulting in death of the organism [53]. The molecular target of compound 5217501 remains unknown. In this study we have shown that Mt-GuaB2 is not the likely target for the antimycobacterial activity of this compound.

Some inhibitor classes evaluated were not active against whole cell *M. smegmatis* while showing potent inhibitory activity against *M. tuberculosis* cultures. A possible explanation may be that *M. smegmatis* is intrinsically resistant to many antibiotics due to the low number of porins which contributes to low permeability through its cell wall [54], [55]. *M. smegmatis* is known to be naturally resistant to compounds of certain classes and shows a resistance to the frontline drugs rifampicin and isoniazid [56]. The lipid bilayer is abnormally thick and shows unusually low fluidity since the trans double bonds present in the α-mycolates decrease fluidity and permeability in *M. smegmatis* [54], [57]. Other than the cell wall permeation barrier, there are additional resistance mechanisms which contribute to intrinsic drug resistance of *M. smegmatis* potentially including multidrug efflux pumps and enzymatic inactivation of drugs [57], [58]. In this study we identified eight hits that potently inhibit *M. smegmatis* growth. Four compounds from each of the Chembridge and Life Chemicals compound set showed good *M. smegmatis* MIC_90_ values ranging from 0.75 µg/ml to 12 µg/ml.

Compound 7759844 has a good selectivity index (SI>23.70), potently inhibited *M. tuberculosis* growth, and inhibits Mt-GuaB2 at nanomolar levels. Hence, the lead compound 7759844 was further evaluated *in vivo* in a mouse model of TB infection based on reported selection criteria [59]. Although the compound was not toxic at 500 mg/kg in a single acute dose MTD determination in normal C57BL/6 mice, the sample was not well tolerated in a more chronic daily dosing regimen of 300 mg/kg for eight days in *M. tuberculosis* infected interferon-γ gene disrupted C57BL/6 (GKO) mice. Beyond these toxicity issues, compound 7759844 was not able to significantly impact disease progression in the GKO efficacy model. Currently, it is not clear what the cause of the observed toxicity might be, although IMPDH is an important enzyme in mammals. Furthermore, *in vivo* metabolic hydrolysis of the arylamide of 7759844 could potentially lead to formation of substituted anilines that are known to undergo metabolic oxidation leading to toxic/genotoxic species. At this point, this highly active class of Mt-GuaB2 inhibitor exemplified by 7759844 remains an excellent lead scaffold for further optimization and development of more active and selective compounds as candidates for new antitubercular drugs. Other classes of inhibitors identified in these studies may also prove fruitful for further drug discovery and development against *M. tuberculosis* IMPDH.

## Supporting Information

Figure S1 **Designed fragments used for searching TB active sets.** The number of TB actives containing each fragment is shown in parenthesis.
(TIF)

Figure S2 **Chemical structures of the Chembridge compound set evaluated in this study.**
(TIF)

Figure S3 **Chemical structures of the LifeChemical compound set.** Included also is compound 1499-0691 which is from ChemDiv.
(TIF)

Figure S4 **SDS-PAGE analysis of purified Mt-GuaB2 fractions.** An aliquot of a series of gradient elutions of increasing imidazole concentrations (150, 200, 300, 400 and 500 mM) were loaded in lanes 1 to 5 of the 12% SDS-PAGE gel after purification through a Ni^2+^ chelate affinity chromatography column. Lane 6 contains the protein molecular weight marker and the numbers towards the right are the molecular masses in kDa. Mt-GuaB2 was visualized after Coomassie blue staining. The arrow in left indicates the purified Mt-GuaB2.
(TIF)

Figure S5 **Determination of** ***K_m_***_** (app)**_ **of substrates IMP (A) and NAD^+^ (B).** Michaelis Menten plot of recombinant Mt-GuaB2 enzyme activity was plotted as a function of varying concentrations of IMP (A) and varying concentrations of NAD^+^ (B). To determine the *K_m_*_ (app)_ values of IMP and NAD^+^ the initial velocity data were fitted to Michaelis Menten equation using nonlinear regression function. The substrate concentration curves were carried out in triplicates. The values represent the mean +/− standard error of three independent experiments.
(TIF)

Figure S6 **Efficacy of Chembridge compound 7759844 in the GKO animal model.** GKO mice were infected with *M. tuberculosis* Erdman strain as described in Materials and Methods. On the fifteenth day of infection, 7759844 (300 mg/kg) and the positive control isoniazid (25 mg/kg) were administered by oral gavage for eight days. Infected untreated mice served as negative control. The mice were sacrificed on day twenty four, the lung and spleen were aspectically removed and homogenates prepared. The number of viable organisms in lungs and spleen were determined by serial ten fold dilutions of homogenates and subsequent plating of dilutions in 7H10 agar plates and incubation at 37°C for 4 weeks. The cfu counts were converted to logarithms and the mean cfu of 7759844 treated mice were compared with untreated mice by one way analysis of variance followed by Dunnett's post test.
(TIF)

Table S1 ***In vivo*** **activity of Isoniazid and Chembridge compound 7759844 in the GKO mouse model.**
(DOC)
