# Supplementary figures and images for: Identification of Novel Mt-Guab2 Inhibitor Series Active against M. tuberculosis
Source: PLoS One. 2012 Mar 29;7(3):e33886. doi: 10.1371/journal.pone.0033886 (PMC3315515; doi:10.1371/journal.pone.0033886)

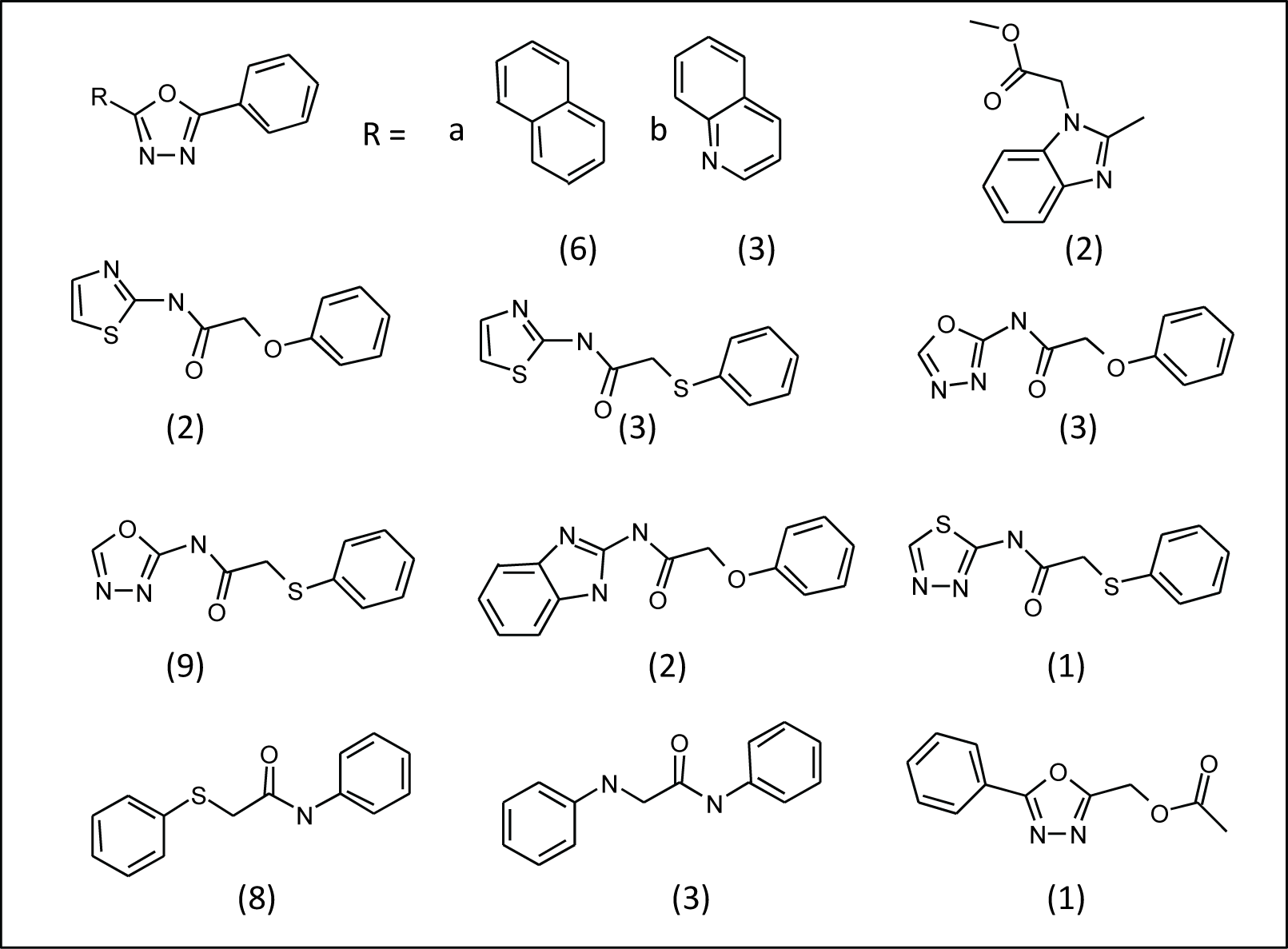

Supplement: Figure S1 — Designed fragments used for searching TB active sets. The number of TB actives containing each fragment is shown in parenthesis. (TIF) [file pone.0033886.s001.tif]

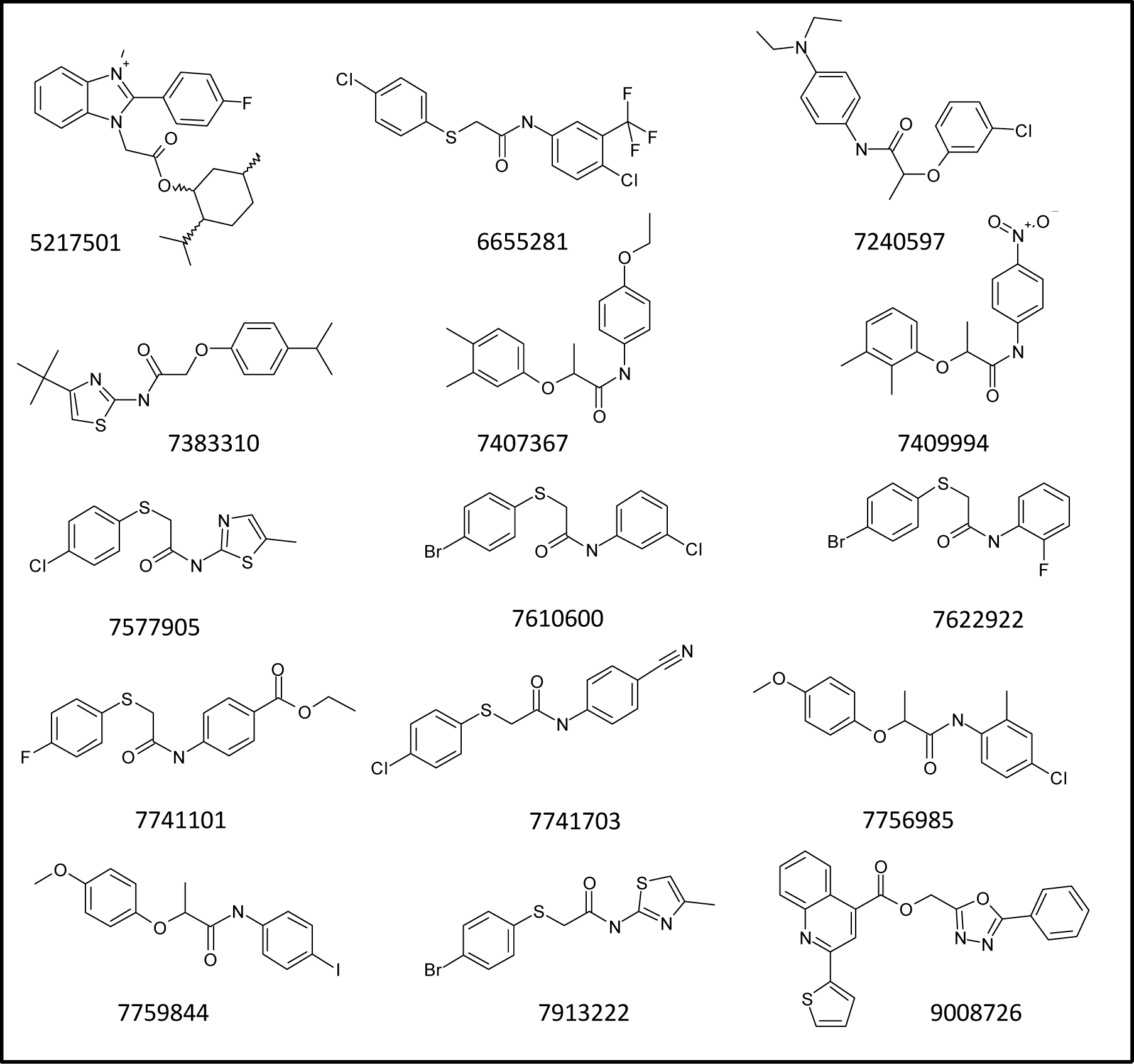

Supplement: Figure S2 — Chemical structures of the Chembridge compound set evaluated in this study. (TIF) [file pone.0033886.s002.tif]

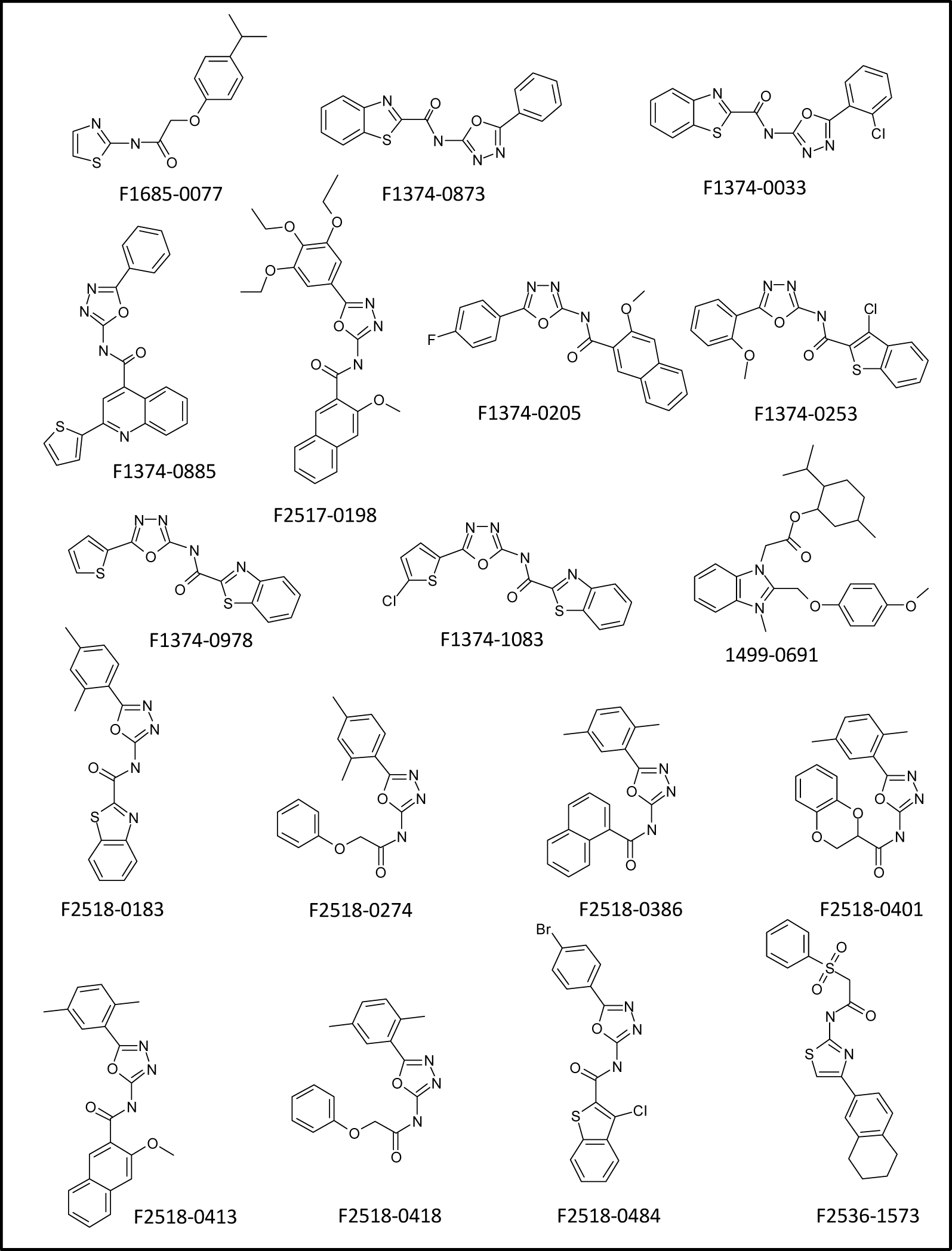

Supplement: Figure S3 — Chemical structures of the LifeChemical compound set. Included also is compound 1499-0691 which is from ChemDiv. (TIF) [file pone.0033886.s003.tif]

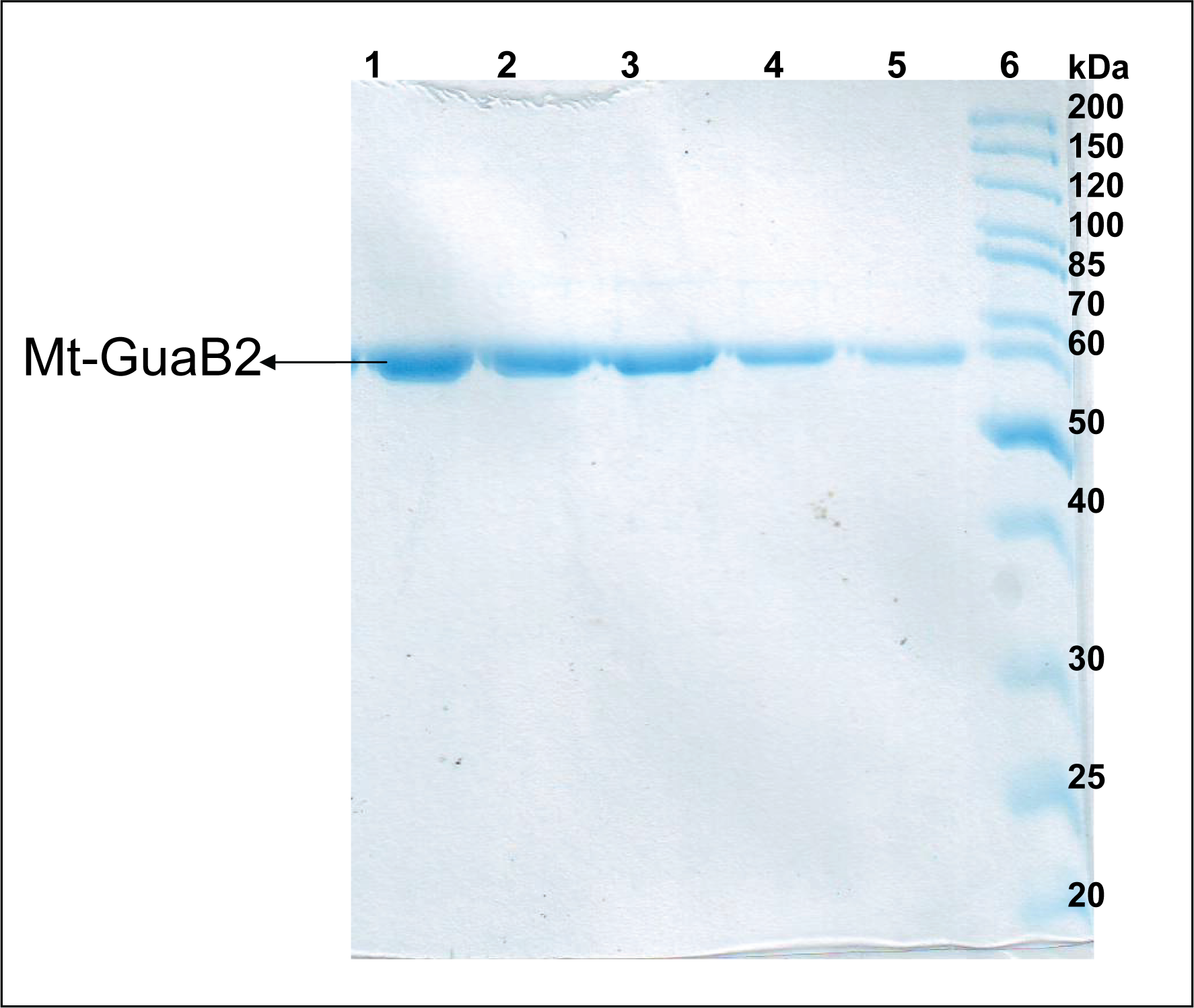

Supplement: Figure S4 — SDS-PAGE analysis of purified Mt-GuaB2 fractions. An aliquot of a series of gradient elutions of increasing imidazole concentrations (150, 200, 300, 400 and 500 mM) were loaded in lanes 1 to 5 of the 12% SDS-PAGE gel after purification through a Ni2+ chelate affinity chromatography column. Lane 6 contains the protein molecular weight marker and the numbers towards the right are the molecular masses in kDa. Mt-GuaB2 was visualized after Coomassie blue staining. The arrow in left indicates the purified Mt-GuaB2. (TIF) [file pone.0033886.s004.tif]

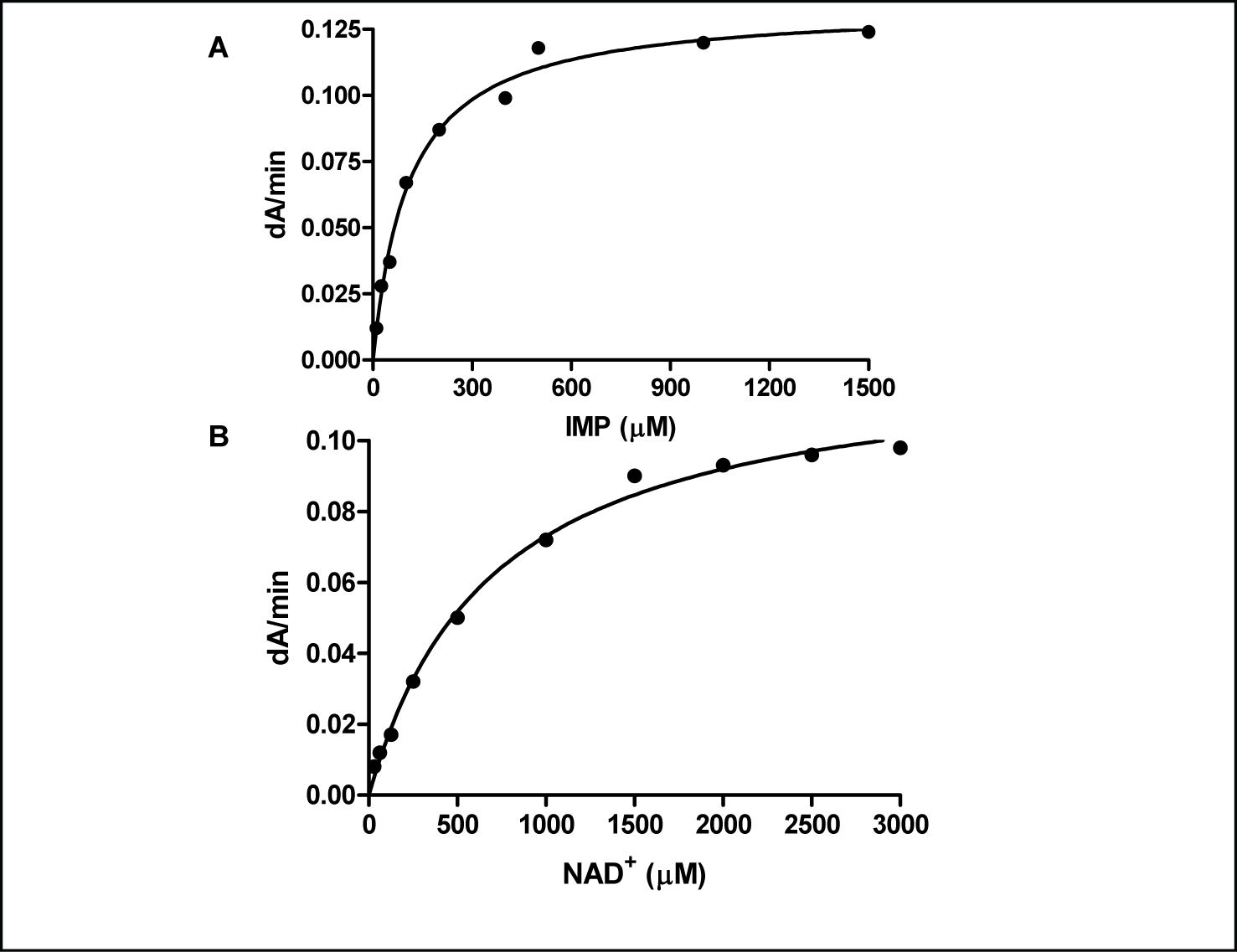

Supplement: Figure S5 — Determination of Km (app) of substrates IMP (A) and NAD+ (B). Michaelis Menten plot of recombinant Mt-GuaB2 enzyme activity was plotted as a function of varying concentrations of IMP (A) and varying concentrations of NAD+ (B). To determine the Km (app) values of IMP and NAD+ the initial velocity data were fitted to Michaelis Menten equation using nonlinear regression function. The substrate concentration curves were carried out in triplicates. The values represent the mean +/− standard error of three independent experiments. (TIF) [file pone.0033886.s005.tif]

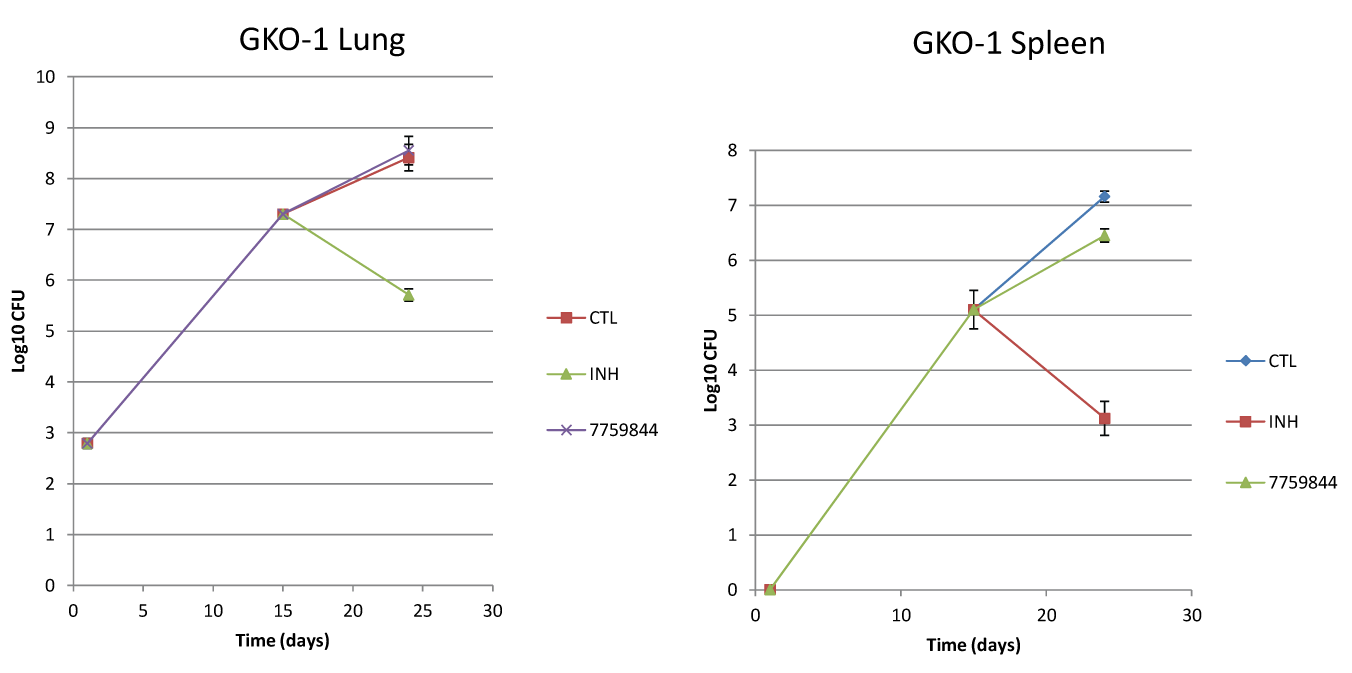

Supplement: Figure S6 — Efficacy of Chembridge compound 7759844 in the GKO animal model. GKO mice were infected with M. tuberculosis Erdman strain as described in Materials and Methods. On the fifteenth day of infection, 7759844 (300 mg/kg) and the positive control isoniazid (25 mg/kg) were administered by oral gavage for eight days. Infected untreated mice served as negative control. The mice were sacrificed on day twenty four, the lung and spleen were aspectically removed and homogenates prepared. The number of viable organisms in lungs and spleen were determined by serial ten fold dilutions of homogenates and subsequent plating of dilutions in 7H10 agar plates and incubation at 37°C for 4 weeks. The cfu counts were converted to logarithms and the mean cfu of 7759844 treated mice were compared with untreated mice by one way analysis of variance followed by Dunnett's post test. (TIF) [file pone.0033886.s006.tif]
