# Supplementary material for: Identification of Novel Mt-Guab2 Inhibitor Series Active against M. tuberculosis
Source: PLoS One. 2012 Mar 29;7(3):e33886. doi: 10.1371/journal.pone.0033886 (PMC3315515; doi:10.1371/journal.pone.0033886)
Supplement: Table S1 — In vivo activity of Isoniazid and Chembridge compound 7759844 in the GKO mouse model. (DOC) [file pone.0033886.s007.doc]

**Table S1**

***In vivo* activity of Isoniazid and Chembridge compound 7759844 in the GKO mouse model**

| Group | Test Group | Organ | Dose | **Mean** | **±** | **SEM** | **Log 10** | Activity |
| --- | --- | --- | --- | --- | --- | --- | --- | --- |
| no. |  |  | in mg/kg/day | **CFU** |  |  | **Protection*** |  |
|  | Untreated - d15 | lung | - | 7.30 | ± | 0.06 |  |  |
|  |  | spleen | - | 5.10 | ± | 0.35 |  |  |
|  |  |  |  |  |  |  |  |  |
| 1 | Untreated - d24 | lung | - | 8.41 | ± | 0.26 |  |  |
|  |  | spleen | - | 7.16 | ± | 0.13 |  |  |
|  |  |  |  |  |  |  |  |  |
| 2 | INH - d24 | lung | 25 | 5.71 | ± | 0.12 | **2.70** |  |
|  |  | spleen |  | 3.12 | ± | 0.31 | **4.04** |  |
|  |  |  |  |  |  |  |  |  |
| 3 | 7759844 | lung | 300 | 8.86 | ± | 0.34 | -0.45 |  |
|  |  | spleen |  | 6.32 | ± | 0.01 | **0.84** | **slightly active** |

Slightly active: statistically better than controls (One-way ANOVA, Dunnett’s)

Active: Log 10 protection (1-2.5)

Very active: Log 10 protection (2.5-4), values for isoniazid
